# High-density Grid Catheter Localizes Eccentric Atrial Flutter to the Left Superior Pulmonary Vein Ridge Through Extreme Isochronal Compression

**DOI:** 10.19102/icrm.2021.120115S

**Published:** 2021-01-15

**Authors:** Dipak Shah, Adam Racette, Jessica Wrobel

**Affiliations:** ^1^Ascension Providence Hospital, Mobile, AL, USA; ^2^Abbott, Chicago, IL, USA

**Keywords:** High-density grid, high-density mapping, left atrial flutter, left atrial ridge, localized reentry

A 75-year-old woman with hypertension and symptomatic drug-refractory paroxysmal atrial fibrillation (AF) elected to undergo AF ablation. Pulmonary vein isolation was performed with a wide antral circumferential ablation. As her esophagus was bordering the left-sided ostia and our left-sided ablation lesion set was more antral, we were also able to isolate her posterior wall. Rapid atrial pacing postablation induced an eccentric atrial flutter (AFL) with a cycle length of 260 ms. Activation mapping with the EnSite Precision™ mapping system with the Advisor™ HD Grid Mapping Catheter, Sensor Enabled™ catheter revealed localized reentry at the left atrial appendage/left superior pulmonary vein ridge. At the critical location, significant compression was seen on the activation color map with a fractionated signal with a duration of 190 ms, essentially encompassing two-thirds of the tachycardia cycle length **(Figure 1 and Video 1)**. Ablation at this region terminated the AFL within a few seconds and the AFL was no longer inducible postablation. This case demonstrates the use of the high-resolution Advisor™ HD Grid catheter using high-density wave technology to analyze orthogonal electrograms at a single location, thereby facilitating rapid identification and treatment of AFL.

## Figures and Tables

**Figure 1:**
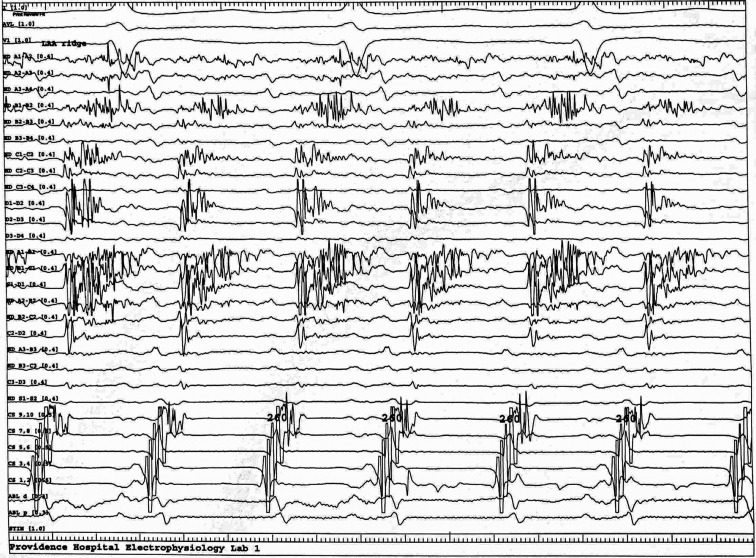
Intracardiac tracing illustrating ~70% of the tachycardia cycle length observed with Advisor™ HD Grid bipole in the left atrial appendage/left superior pulmonary vein ridge.

**Video 1:** Propagation map of left AFL demonstrating slowed conduction in the left atrial appendage/left superior pulmonary vein ridge.

